# Association of gestational diabetes mellitus and negative modulation of the specific humoral and cellular immune response against *Toxoplasma gondii*

**DOI:** 10.3389/fimmu.2022.925762

**Published:** 2022-09-20

**Authors:** Ana Carolina de Morais Oliveira-Scussel, Paula Tatiana Mutão Ferreira, Renata de Souza Resende, Cristhianne Molinero Ratkevicius-Andrade, Angelica de Oliveira Gomes, Marina Carvalho Paschoini, Fernanda Bernadelli De Vito, Thaís Soares Farnesi-de-Assunção, Marcos Vinícius da Silva, José Roberto Mineo, Denise Bertulucci Rocha Rodrigues, Virmondes Rodrigues

**Affiliations:** ^1^ Laboratory of Immunology, Institute of Biological and Natural Sciences, Department of Microbiology, Immunology and Parasitology, Universidade Federal do Triângulo Mineiro (UFTM), Uberaba, Minas Gerais, Brazil; ^2^ Laboratory of Cellular Interactions, Institute of Biological and Natural Sciences, Department of Structural Biology, Universidade Federal do Triângulo Mineiro (UFTM), Uberaba, Minas Gerais, Brazil; ^3^ Institute of Health Sciences, Department of Obstetricy, Universidade Federal do Triângulo Mineiro (UFTM), Uberaba, Minas Gerais, Brazil; ^4^ Laboratory of Hematology and Hemotherapy, Institute of Health Sciences, Universidade Federal do Triângulo Mineiro (UFTM), Uberaba, Minas Gerais, Brazil; ^5^ Laboratory of Parasitology, Institute of Biological and Natural Sciences, Department of Microbiology, Immunology and Parasitology, Universidade Federal do Triângulo Mineiro (UFTM), Uberaba, Minas Gerais, Brazil; ^6^ Laboratory of Immunology “Dr. Mário Endsfeldz Camargo”, Institute of Biomedical Sciences, Universidade Federal de Uberlândia (UFU), Uberlândia, Minas Gerais, Brazil; ^7^ CEFORES, Universidade Federal do Triângulo Mineiro (UFTM), Uberaba, Minas Gerais, Brazil

**Keywords:** *Toxoplasma gondii*, gestational diabetes mellitus (GDM), humoral immune response, cellular immune response, toxoplasmosis, pregnancy

## Abstract

In order to evaluate and compare the specific immune response of pregnant women (PW) chronically infected with *Toxoplasma gondii*, with and without gestational diabetes mellitus (GDM), and the humoral response of their respective newborns (NB), the study was carried out on 81 PW (34 GDM and 47 controls) from whose medical records the results of the oral glucose tolerance test (OGTT) were obtained, and blood samples were collected at the third trimester of pregnancy; also, on 45 NBs (20 GDM and 25 controls) from whom umbilical cord blood samples were obtained. Humoral immunity was analyzed by measuring anti-*T. gondii* total IgG, IgG subclasses and IgG avidity. To evaluate cellular immunity, peripheral blood mononuclear cells (PBMC) from 32 PW (16 GDM and 16 controls) were cultured, supernatant cytokines were determined, and flow cytometry was performed to analyze the expression at lymphocytes of surface molecules, cytokines and transcription factors. All PW and NBs were positive for total IgG, and the prevalent subclass was IgG1. There was a negative correlation between the OGTT glycemia of PW and the levels of total IgG, IgG1 and IgG avidity. The IgG avidity of the GDM group was significantly lower than the control group. Patients from the GDM group had a higher number of T lymphocytes expressing markers of cell activation and exhaustion (CD28 and PD-1). In the presence of *T. gondii* soluble antigen (STAg) the amount of CD4^+^ T cells producing IFN-γ, IL-10 and IL-17 was significantly lower in the GDM group, while there was no difference between groups in the number of CD4^+^ CD25^High^FOXP^3^+LAP+ functional Treg cells. Additionally, under STAg stimulus, the secretion of IL-17, IL-4, TNF and IL-2 cytokines at PBMCs culture supernatant was lower in the GDM group. In conclusion, there was a correlation between the increase in blood glucose and the decrease in levels of anti-*T. gondii* antibodies, associated with the decreased IgG avidity in patients who develop GDM. Also, the GDM group had decreased immune responses in Th1, Th2 and Th17 profiles, suggesting an association between GDM and the negative modulation of the humoral and cellular immune responses against *T. gondii*.

## Introduction

Toxoplasmosis is a disease caused by the intracellular parasite *Toxoplasma gondii* and considered one of the most common zoonoses in the world (1–3). In Brazil, the seroprevalence in humans is very high, and varies between 21% and 97%, depending on the studied group (4). The investigation of this infection is important in public health due to its occurrence in severe clinical forms in newborns with congenital infection and in immunocompromised individuals (5–7).

Fetal infection occurs through transplacental passage; when the mother acquires the infection during pregnancy, or when it is reinfected by a new strain of *T. gondii* (8, 9) or, less commonly, when chronically infected women have an important immunocompromise (10), since reactivation of the infection may occur in immunocompromised patients (11–15). Therefore, even the pregnant woman (PW) considered immune to infection by *T. gondii* (IgG-positive) presents risks of vertical transmission of the parasite to the fetus. In Brazil, several studies have reported the high prevalence of *T. gondii* in IgG-positive PW (16–26).

There are several studies about toxoplasmosis in PW and in immunosuppressed patients, however, there is a lack of data in the literature regarding the possibility of the reactivation of the infection in PW with gestational diabetes mellitus (GDM).

GDM is a type of diabetes mellitus characterized by insulin resistance and glucose intolerance that begins during pregnancy (27–29). Its prevalence varies from 0.6% to 25%, depending on the socioeconomic level, the ethnic group studied and the diagnostic criteria used, and it is considered the most frequent metabolic comorbidity in pregnancies (29–32).

Literature data describing a relationship between GDM and toxoplasmosis are restricted to serology (33, 34). There are few studies that have investigated the role of innate and adaptive immune cells in GDM pathophysiology (27), and there is still no data regarding the pattern of immune response present in GDM’s association with toxoplasmosis. Due to the importance of managing better strategies for the prevention, diagnosis and treatment of toxoplasmosis and reduction of congenital transmission, this study was conducted in order to analyze the humoral and cellular immune response of *T. gondii* seropositive PW who developed GDM, and to evaluate the humoral immune response of their newborns (NB).

## Materials and methods

### Subjects

The study was carried out on 81 PW in the third trimester of pregnancy who were receiving prenatal care at the Gynecology and Obstetrics Clinic of the Clinical Hospital, Universidade Federal do Triângulo Mineiro (CH-UFTM), Minas Gerias, Brazil. Inclusion criteria of the study consisted of women over 18 years, with gestation of 29 weeks or more, anti-*T. gondii* IgG positive and IgM negative. Exclusion criteria consisted of PW with negative or undetermined serology for toxoplasmosis, patients with type 1 or 2 diabetes mellitus (DM1 or DM2), patients who had cancer or some chronic transmissible disease or infectious contagious comorbidities (e.g., HIV, HCV, HBV, syphilis). The study was reviewed and approved by Universidade Federal do Triângulo Mineiro’s Ethical Committee (Protocol number: 1.870.741) and written informed consent was obtained from all patients. The study was conducted in accordance with the World Medical Association’s Declaration of Helsinki.

### Biological samples

From each patient, peripheral blood samples were collected (tubes with and without anticoagulant) to obtain serum and peripheral blood mononuclear cells (PBMCs) for the work experiments. Also, all PW’s data regarding age, education, family income and gestational age were collected *via* interview. In addition, a medical record analysis was carried out to obtain the result of anti-*T. gondii* serology, the result of the oral glucose tolerance test (OGTT), the medical diagnosis of gestational diabetes mellitus and information about comorbidities.

Additionally, blood samples of NBs whose mothers were included in the work and who were born at CH-UFTM were obtained from the umbilical cord immediately after delivery. The cord blood samples were used to obtain NB plasma to carry out serological experiments.

### Groups and experimental design

From the medical diagnosis of GDM, according to the criteria recommended by the Brazilian Diabetes Society (28), the 81 patients were divided into two groups. Thirty-four PW who developed gestational diabetes mellitus (GDM group) and 47 PW without diabetes (control group). The work also involved samples from 45 NBs (20 GDM and 25 control) of these women. The reduction in the number of NBs in relation to the number of PW is due to either the delivery not having been performed at the CH-UFTM or the mother’s lack of authorization to use their umbilical cord blood.

The evaluation of the humoral immune response of 81 PW and 45 NBs was carried out by ELISA assays for measurements of IgG anti-*T. gondii* antibodies, their subclasses and IgG avidity.

Out of the 81 PW, 32 (16 GDM and 16 controls) were also involved in the cellular immune response evaluation, which was done by the isolation and cultivation of each patient’s PBMCs for 96 hours under polyclonal stimulus and the specific stimulus of *T. gondii*. At the end of this time, culture supernatants were used to measure cytokines (Th1, Th2 and Th17 profiles), while the cells were used to analyze surface molecules, transcription factors and cytokines expressed in cells in order to assess cell activation and exhaustion, Treg cell phenotype and cytokine expression by CD4^+^ T lymphocytes.

### Parasites and antigens

*T. gondii* RH strain tachyzoites were maintained by serial passage in cell culture for 48–72 hours, using HeLa cell lines (ATCC/CCL-2; American Type Culture Collection, Manassas, VA, USA), as described above (35). Briefly, cells were inoculated with tachyzoites of *T. gondii* and maintained by serial passages in an RPMI medium with 5% fetal bovine serum. Free parasites were collected and washed three times with phosphate buffered saline (PBS, pH 7.2 – 900 x g for 10 minutes at 4°C) and, after addition of a protease inhibitor cocktail (Complete Ultra tablets, Mini, Easypack, Roche, USA), lysates were obtained by repeated freezing and thawing (10 cycles), sonication, and centrifugation at 10,000 × g for 30 minutes at 4°C. After supernatant recovery, total protein was estimated by the Bradford method (36) and the aliquots were stored as soluble tachyzoite antigens (STAg) at -80°C until later use. The STAg used in the cell culture experiments was prepared similarly, without addition of the protease inhibitor cocktail, and the supernatant recovery was filtered through a membrane with a 0.22 μm pore.

### Indirect ELISA to detect IgG anti-*T. gondii* antibodies

To detect IgG antibodies in samples of PW and NBs, ELISA was performed as previously described (37) with modifications. Wells of high affinity microtiter plates (Nunc MaxiSorp™, Thermo Fisher Scientific, Waltham, USA) were coated with 50 μL of STAg (10 μg/mL) in 0.06 M carbonate buffer (pH 9.6) and incubated overnight at 4°C. The plates were washed three times with PBS-Tween 0.05% (PBS-T) and blocked with PBS-T plus 5% of nonfat powdered milk (Molico, Nestlé, São Paulo, Brazil) (PBS-T-M5%) for one hour at room temperature. Samples of serum/plasma (1:64 in PBS-T-M5%) were incubated for two hours at 37°C. After washing, the plates were incubated with anti-human IgG antibodies labeled with peroxidase (DAKO North America, Fisher Scientific, Carpinteria, USA – 1:2000 in PBS-T-M5%) for one hour at 37°C. For IgG subclass, plates were incubated with each biotinylated detection antibody (BD Biosciences, San Jose, USA – IgG1, IgG2 and IgG3 1:1000 in PBS-T-BSA1%, IgG 1:2000 in PBS-T-BSA1%) flowed by the addition of streptavidin-peroxidase (BD Biosciences, San Jose, USA) diluted 1: 1000 in PBS-T-BSA1%. The reactions were revealed by adding enzymatic substrate (TMB - 3,3′,5,5′-Tetramethylbenzidine – Merck Millipore, Darmstadt, Germany), stopped with 1M phosphoric acid and optical densities (OD) were determined at 450 nm. Two positive controls and three negative controls were included in each plate in order to calculate the cut-off, which was established as the mean OD values for negative controls plus three standard deviations. Results were expressed as ELISA index (EI) as follows: EI = OD sample/OD cut-off, where values of EI ≥ 1.2 were considered positive.

### IgG avidity ELISA assay

Avidity ELISA assay was performed to detect the avidity of total IgG anti-*T. gondii* antibodies in samples of PW and NBs. After incubation with serum samples in duplicated wells of microtiter plates, one well was washed with PBS-T (urea-) and the other washed with PBS-T containing 8 M of urea (urea+) for 15 minutes at room temperature. After washing with PBS-T, the wells were submitted to the ELISA assays as described above. The results were expressed in avidity index (AI) as AI = (well EI urea+/well EI urea−) x 100. AI < 30% corresponds to antibodies of low avidity, 30–60% AI corresponds to antibodies of intermediate avidity, while AI > 60% corresponds to antibodies of high avidity.

### Cell culture

Ficoll-Hypaque (d = 1.077 g/mL; Histopaque-1077, Sigma-Aldrich) was used to separate, by density gradient, the PBMCs of the PW. The cells were resuspended in an RPMI medium supplemented with 10% SFB, and the number of cells determined by counting in a Neubauer chamber, using Turk’s liquid. Then, the PBMCs (2 x 106 cells/well) of each PW were cultured in 24-well culture plates (BD Falcon^®^ - Bedford, MA, USA) under three different conditions: without stimulus (only with RPMI medium supplemented with 10% SFB); polyclonal stimulus (1μg/mL of anti-CD3 antibodies and 0.25μg/mL of anti-CD28 - BD Biosciences, USA); and the specific stimulus of *T. gondii* (5μg/mL of STAg). The cells were incubated with 5% CO2 at 37 °C for 96 hours. After 4 days, the culture supernatants were collected and stored at -80°C until cytokine analysis, and the cells were immediately used for flow cytometry phenotyping.

### Flow cytometry phenotyping

In the last 6 hours of culture, PBMCs were incubated with Golgistop™ solution (BD Biosciences, USA). At the end of the 96 hours of culture, the cells were collected, centrifuged (400 x g, 4°C, 5 minutes) and incubated in PBS supplemented with 10% AB human serum for 30 minutes. Then, the cells of each culture condition were divided into four distinct tubes to carry out all the markings: tube one (blank - without the addition of antibodies); tube two (anti-CD8-BB515, anti-PD-1-PE, anti-CD4-PE-Cy7 and anti-CD28-APC); tube three (anti-CD25-FITC, anti-Foxp3-PE, anti-CD4-PE-Cy7 and anti-LAP-Alexa Fluor 647); and tube four (anti-IL17-Alexa Fluor 488, anti-IL10-PE, anti-CD4-PE-Cy7 and anti-IFNγ-Alexa Fluor 647) (BD Biosciences, USA). The antibodies were used according to the manufacturer’s recommendations. The antibodies directed to cell surface molecules were added first, incubated for 30 minutes at 4°C and protected from light. After washing, the cells were fixed and permeabilized with 100μL of Cytofix/Cytoperm (BD Biosciences, USA), for 30 minutes at 4°C. Then, the cells were washed with BD Perm/Wash buffer (BD Biosciences, USA) and incubated with antibodies directed to intracellular molecules for 30 minutes at 4°C and protected from light. At the end, the cells were resuspended in 100μL of 0.5% paraformaldehyde and stored at 4°C protected from light until analysis in the flow cytometer. In parallel to the labeled tubes, control isotypes were used. The acquisition of 50,000 events was performed using a FACSCantoII cytometer (BD Biosciences, USA) using the Diva 6.0 program. Data analysis was performed using the FlowJo 10.6.1 program.

### Cytokines in the culture supernatant

The IL-2, IL-4, IL-6, IL-10, TNF, IFN-γ and IL-17A cytokines present in the culture supernatant were measured using the cytometric bead array (CBA) technique, using the BD CBA Human Th1/Th2/Th17 Cytokine Kit (Catalog: 560484), according to the manufacturer’s instructions. Data were acquired using a FACSCalibur cytometer (BD Biosciences, USA), using the Cell Quest program (BD Biosciences, USA). The FCAP Array 2.0 software (BD Biosciences, USA) was used to analyze the results and the cytokine concentrations calculated from the standard curve. The detection limit for IL-2, IL-4, IL-6, IL-10, TNF, IFN-γ and IL-17 cytokines were 2.6; 4.9; 2.4; 4.5; 3.8; 3.7 and 18.9 pg/ml, respectively.

### Statistical analysis

Statistical analysis was carried out using the GraphPad Prism 7.0 (GraphPad Software Inc., San Diego, USA). Differences between the glycemia of OGTT and levels of total IgG of PW and NBs were analyzed using unpaired *t*-test (between the GDM and control groups) and paired *t*-test (within each group). All other variables studied were analyzed using Mann-Whitney U test (between the GDM and control groups) and using Wilcoxon test (within each group). The Spearman correlation test was used to correlate the OGTT values with the levels of total IgG, IgG1 and IgG avidity of PW. For all tests, values of *p* < 0.05 were considered statistically significant.

## Results

### Sample characterization

The 81 PW involved in this work were divided into GDM and control groups, according to the medical diagnosis of GDM and the results of OGTT. All were in the third trimester of gestation with mean gestational age of 35.5 (GDM) and 35 (control) weeks (**Table 1**).

**Table 1:** Sample characterization, number and percentage of pregnant women with and without Gestational Diabetes Mellitus (GDM) and their newborns, in relation to seropositivity for anti-*T. gondii* antibodies.

	Pregnant women		Newborn	
Groups	Controln=47	GDMn=34	Controln=25	GDMn=20
**Gestational age (weeks), mean**	35	35.5	–	–
**Age (years), mean**	27.4	31.5	–	–
**OGTT glycemia (mg/dL), mean**				
Fasting	79.2	101.0	–	–
One hour	114.8	170.1	–	–
Two hours	101.1	147.2	–	–
**Positive samples, number (%)**				
Total IgG	47 (100)	34 (100)	25 (100)	20 (100)
IgG1	46 (97,9)	33 (97,1)	25 (100)	20 (100)
IgG2	2 (4,3)	1 (2,9)	0	1 (5)
IgG3	3 (6,4)	2 (5,9)	1 (4)	0
IgG4	8 (17)	8 (23,5)	3 (12)	4 (20)

The GDM group’s glycemic values of fasting and one hour and two hours after ingestion of 75g of dextrose (mean: 101.0; 170.1 and 147.2 mg/dL, respectively) were statistically higher (*p* < 0.0001, unpaired *t*-test) than the values of the control group (mean: 79.2; 114.8 and 101.1 mg/dL, respectively) (**Table 1**).

The GDM group involved 34 PW with mean age of 31.5 years (median: 31 years, range: 21–43 years, standard deviation: 6.1). The control group involved 47 PW with mean age of 27.4 years (median: 26 years, range: 18–41 years, standard deviation: 5.5) (**Table 1**). The mean age of diabetic PW was significantly higher (*p* = 0.0022). For both groups, the level of education and family income were similar.

### Humoral immune response specific to *T. gondii*

The number and percentage of PW and NBs positive for total IgG, IgG1, IgG2, IgG3 and IgG4 anti-*T. gondii* antibodies are shown in **Table 1**. For all positive results of the NBs, the respective mother was also positive for the respective immunoglobulin.

**Figure 1:**
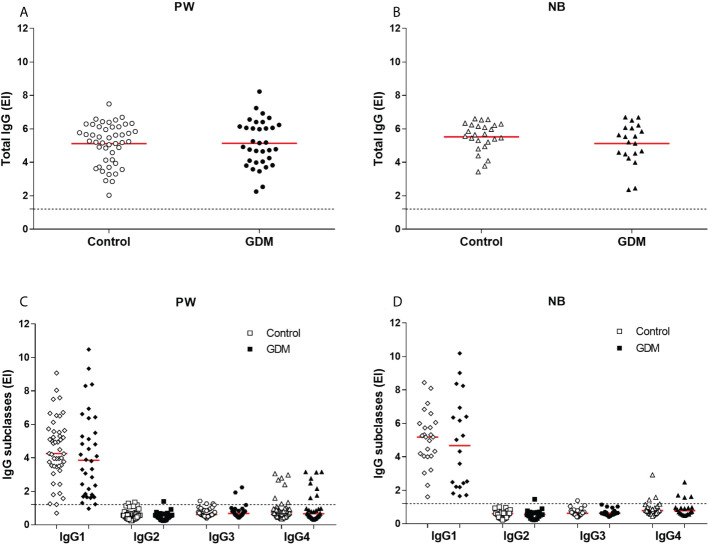
Anti-*T. gondii* antibody levels. **(A)** Total IgG in serum samples from 81 pregnant women (34 GDM and 47 controls) and **(B)** 45 newborns (20 GDM and 25 controls). **(C)** IgG1, IgG2, IgG3 and IgG4 in serum samples from 81 pregnant women (34 GDM and 47 controls) and **(D)** 45 newborns (20 GDM and 25 controls). The red line represents the mean (total IgG) and median (subclasses) of the values. The dashed line represents the cut-off value (EI:1,2). PW, Pregnant women; NB, Newborn; EI, ELISA Index; GDM, Gestational diabetes mellitus.

The levels of total IgG anti-*T. gondii* antibodies were positive for all samples (**Figure 1**), but there were no statistical differences between the values of the PW from the GDM group (mean: 5.13) and from the control group (mean: 5.11), *p* = 0.9567 (**Figure 1A**); nor between the values of NBs from the GDM group (mean: 5.12) and from the control group (mean: 5.51), *p* = 0.2153 (**Figure 1B**).

There were also no statistical differences between the levels of IgG subclasses antibodies specific to the parasite, when comparing the values between the groups of PW (**Figure 1C**) and between the groups of NBs (**Figure 1D**). The median values for PW from the GDM and control groups were, respectively: IgG1 (3.85 and 4.26, *p* = 0.2738), IgG2 (0.52 and 0.57, *p* = 0.1054), IgG3 (0.67 and 0.61, *p* = 0.7016) and IgG4 (0.66 and 0.76, *p* = 0.6736). The median values for NBs from the GDM and control groups were IgG1 (4.67 and 5.18, *p* = 0.6146), IgG2 (0.55 and 0.60, *p* = 0.7730), IgG3 (0.63 and 0.62, *p* = 0.8078) and IgG4 (0.78 and 0.78, *p* = 0.8965), respectively.

It is possible to observe that, among the subclasses, there was a predominance of IgG1 in the response against *T. gondii* in both samples (PW and NBs), and that the number of positive patients for the other subclasses was small, mainly for IgG2 and IgG3 (**Table 1** and **Figure 1**).

**Figure 2:**
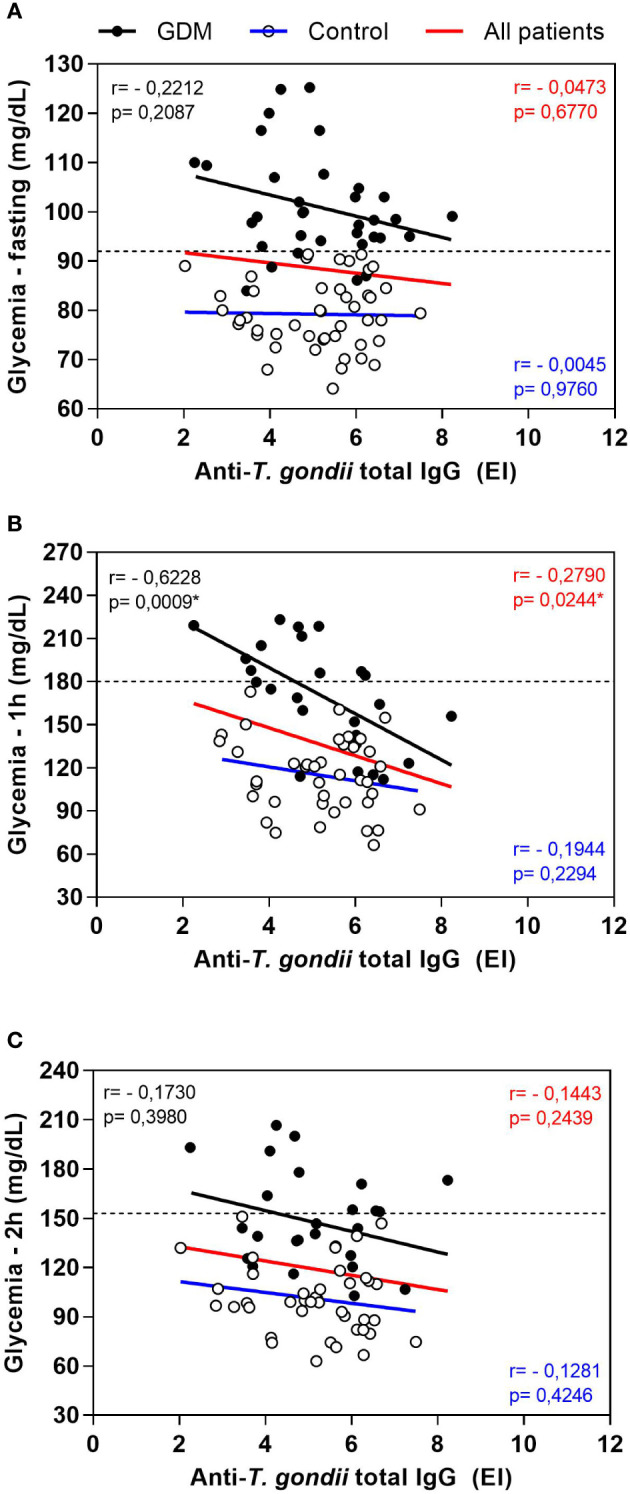
Correlation between oral glucose tolerance test (OGTT) blood glucose values and anti-*T. gondii* total IgG levels of pregnant women, from the GDM group, control group and all patients. **(A)** Fasting blood glucose, **(B)** glycemia one hour after dextrose and **(C)** glycemia two hours after dextrose. The dashed lines represent the cut-off values for the diagnosis of gestational diabetes mellitus (Fasting > 92 mg/dL; one hour ≥ 180 mg/dL, two hours ≥ 153 mg/dL). *Statistically significant data, Spearman correlation test. EI: ELISA Index. GDM: Gestational diabetes mellitus.

The total IgG anti-*T. gondii* antibodies levels were correlated with the OGTT glycemia values of the PW, this correlation was performed separately in the GDM and control groups, considering all patients at the same time (**Figure 2**). This analysis showed a negative correlation between the two parameters, which meant that the higher the glycemic indexes, the lower the levels of total IgG anti-*T. gondii* antibodies. This negative correlation was observed for the three OGTT (fasting, **Figure 2A**; one hour, **Figure 2B**; and two hours, **Figure 2C**), and statistically significant values were found in the glycemia of one hour after ingesting the dextrose (*r* = -0.2790, *p* = 0.0244 for all patients; *r* = -0.6228, *p* = 0.0009 for the GDM group; **Figure 2B**).

The same results were found for the correlation between the levels of IgG1 anti-*T. gondii* antibodies and the OGTT glycemia values (data not shown). At the time of fasting, the negative correlation was not statistically significant. At one hour, the negative correlation was significant for the GDM group (*r* = -0.5746, *p* = 0.0027) and for all patients together (*r* = -0.3821, *p* = 0.0017). At two hours, the negative correlation was also significant when all patients were analyzed together (*r* = -0.2802, *p* = 0.0216).

**Figure 3:**
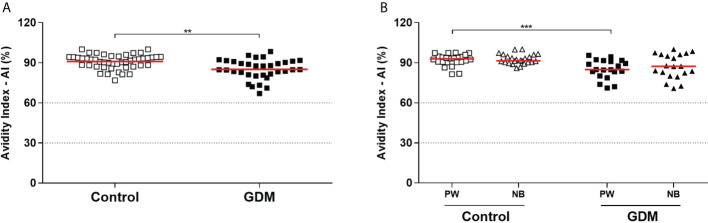
Anti-*T. gondii* total IgG antibodies avidity index, expressed as a percentage (%). **(A)** Samples from 81 pregnant women (34 GDM and 47 controls). **(B)** Samples from 45 pregnant women (20 GDM and 25 controls) and their respective newborns. Dotted lines demarcate areas that represent low avidity (0–30%), indeterminate avidity (30–60%), and high avidity (> 60%). The red line represents the median of the values. Statistically significant data, *p* < 0.05 **Unpaired *t*-test ***Mann-Whitney U test. PW, Pregnant women; NB, Newborn; GDM, Gestational diabetes mellitus.

Additionally, the IgG avidity of PW in the GDM group (mean: 85.1%) was significantly lower than the control group (mean: 90.94%), *p* < 0.0001 (**Figure 3A**). This lower avidity observed in GDM group did not represent a clinically relevant decrease. Furthermore, difference, in the IgG avidity did not occur between the two groups of NBs (91.53% control vs 87.31% GDM, *p* = 0.0799), nor when the NBs were compared with their respective mothers (84.87% mother vs 87.31% NB, *p* = 0.1650 for the GDM group, and 92.88% mother vs 91.53% NB, *p* = 0.9218 for the control group) (**Figure 3B**).

A correlation between the IgG avidity values and the OGTT glycemia values of the PW was also performed (data not shown), which showed a significant negative correlation in the three OGTT (fasting, one hour and two hours) when considering all patients together (*r* = -0.3497, *p* = 0.0015; *r* = -0.5005, *p <*0.0001; *r* = -0.3442, *p* = 0.0043, respectively). In addition, at one hour, the negative correlation was also significant in the GDM group (*r* = -0.5108, *p* = 0.0091). Therefore, the higher the glycemic index, the lower the avidity of IgG anti-*T. gondii* antibodies.

### Cellular immune response

#### T lymphocyte activation and exhaustion

**Figure 4:**
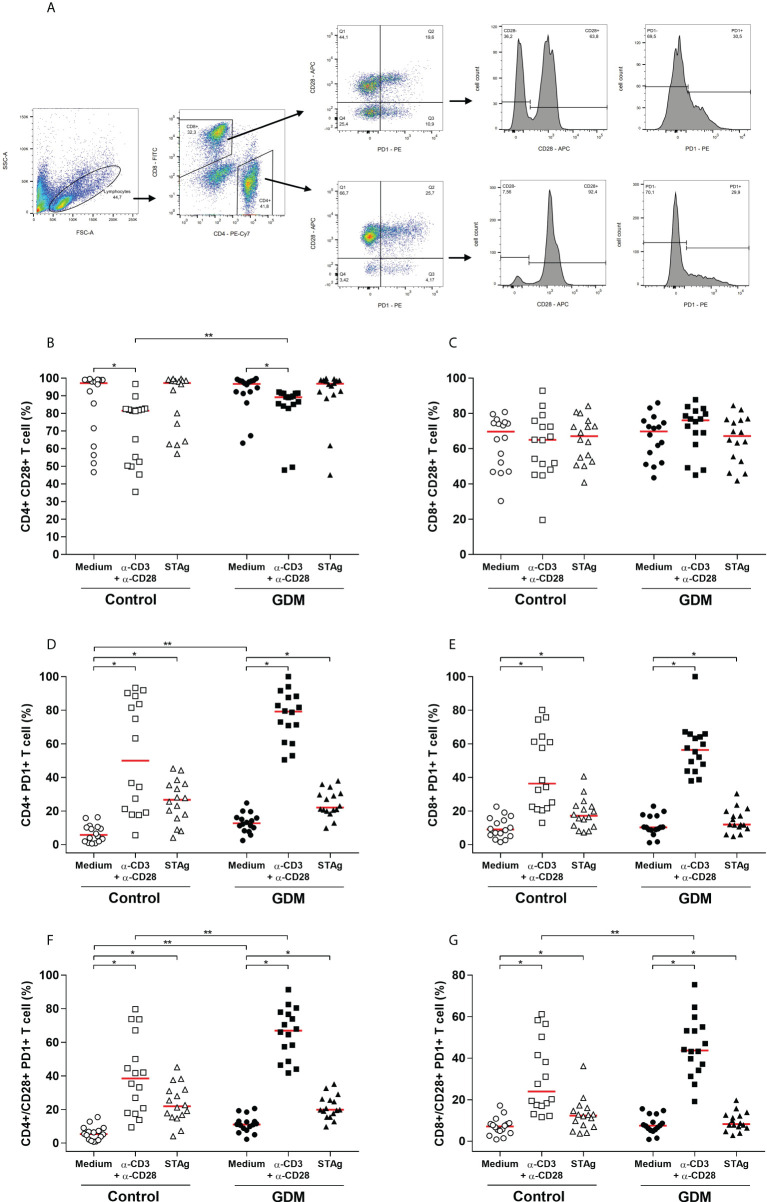
Activation and exhaustion of T lymphocytes, evaluated by the expression of CD28 and PD-1 cell surface receptors. **(A)** Gating strategy employed. Percentage (%) of **(B)** CD4^+^CD28^+^, **(C)** CD8^+^CD28^+^, **(D)** CD4^+^PD-1^+^, **(E)** CD8^+^PD-1^+^, **(F)** CD4^+^/CD28^+^PD-1^+^, **(G)** CD8^+^/CD28^+^PD-1^+^ T cell, acquired by flow cytometry of PBMC cells from 32 pregnant women (16 GDM and 16 controls) after 96 hours culture in three different conditions (medium, polyclonal α-CD3/α-CD28 stimulus and specific STAg stimulus). The red line represents the median of the values. Statistically significant data, *p* < 0.05 *Wilcoxon **Mann-Whitney U test. GDM, Gestational diabetes mellitus.

The CD28 co-receptor and the programmed cell death-1 receptor (PD-1), which are markers of cell activation and exhaustion, respectively, were analyzed by flow cytometry in CD4^+^ and CD8^+^ T lymphocytes and the gating strategy is shown in **Figure 4A**.

When PBMCs were stimulated with anti-CD3/anti-CD28, there was a significant increase in CD8^+^ T lymphocytes and a significant decrease in CD4^+^ T lymphocytes, in relation to cells cultured with only a medium, both in the GDM group (*p* = 0.0004 and *p* < 0.0001, respectively) and in the control group (*p* < 0.0001 and *p* < 0.0001, respectively). When compared between the two PW groups, there was no significant difference in the amount of CD4^+^ T cells in any of the three culture conditions. However, PW from the GDM group had higher amounts of CD8^+^ T cells compared to PW from the control group, and this difference was significant both in the medium culture condition (*p* = 0.0253) and under the specific *T. gondii* antigen stimulation (*p* = 0.0378) (data not shown).

Analysis of the expression of the CD28 co-receptor revealed that high amounts of CD4^+^ T lymphocytes expressed this receptor, but in the GDM group (*p* < 0.0001) and the control group (*p* = 0.0092), less CD4^+^ CD28^+^ T cells were detected when PBMCs were stimulated with anti-CD3/anti-CD28 in relation to the culture with only a medium. Also, when comparing the two groups of PW, it was observed that, with anti-CD3/anti-CD28 stimulus, the GDM group expressed more CD4^+^ CD28^+^ T cells than the control group (*p* = 0.0025) (**Figure 4B**). In addition, high amounts of CD8^+^ T lymphocytes also expressed CD28, and there was no change in this quantity under any of the three culture conditions, nor between the two groups of PW studied (**Figure 4C**).

Regarding PD-1, the two stimuli used in culture significantly increased the number of CD4^+^ T cells expressing PD-1, in both groups of PW (*p* < 0.0001). The comparison between the groups revealed that, in the unstimulated cells, the GDM group had more CD4^+^PD-1^+^ T cells than the control group (*p* = 0.0038) (**Figure 4D**). Likewise, it was observed that the polyclonal and specific stimuli of *T. gondii* significantly increased the number of CD8^+^ T cells expressing PD-1, in both groups (GDM (*p* < 0.0001 and *p* = 0.0027, respectively) and control (*p* < 0.0001 and *p* = 0.0021, respectively)). However, there was no difference in the amount of CD8^+^PD-1^+^ T lymphocytes between the two groups of patients (**Figure 4E**).

The CD28 and PD-1 double labeling in lymphocytes was analyzed and in both groups of PW, the anti-CD3/anti-CD28 and STAg stimuli significantly increased the amount of CD4^+^CD28^+^PD-1^+^ T cells (*p* < 0.0001) (**Figure 4F**) and CD8^+^CD28^+^PD-1^+^ T cells (*p* < 0.05) (**Figure 4G**). Additionally, under polyclonal stimulation, the number of CD4^+^ and CD8^+^ T cells expressing the two receptors was significantly higher in the GDM group (*p* = 0.0019 and *p* = 0.0211, respectively) (**Figures 4F, G**). In the GDM group, the number of CD4^+^CD28^+^PD-1^+^ T cells was also significantly higher even in the absence of stimulation (*p* = 0.0022) (**Figure 4F**).

#### Intracellular production of cytokines by CD4^+^ T cells

**Figure 5:**
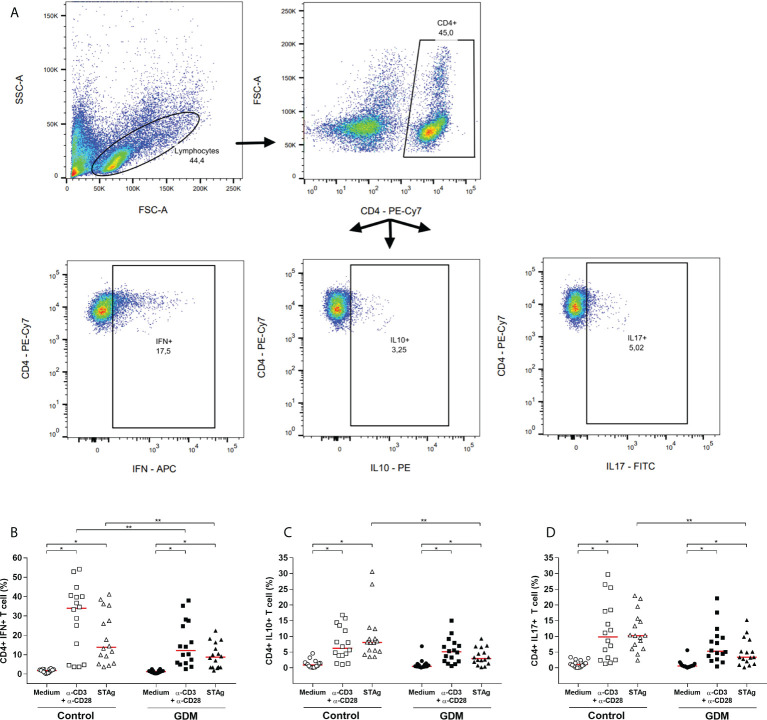
Intracellular production of cytokines by CD4^+^ T lymphocytes. **(A)** Gating strategy employed. Percentage of CD4^+^ T cells producing **(B)** IFN-γ, **(C)** IL-10, **(D)** IL-17, which were acquired by flow cytometry of PBMC cells from 32 pregnant women (16 GDM and 16 controls) after 96 hours culture in three conditions (medium, polyclonal α-CD3/α-CD28 stimulus and specific STAg stimulus). The red line represents the median of the values. Statistically significant data, p<0.05 *Wilcoxon **Mann-Whitney. GDM, Gestational diabetes mellitus.

Intracellular production of IFN-γ, IL-10 and IL-17 cytokines by PW CD4^+^ T cells was assessed by flow cytometry, and the gating strategy is shown in **Figure 5A**.

In both the GDM and control groups, in comparison with the medium-only culture condition, the anti-CD3/anti-CD28 and the STAg stimulus significantly increased the percentage of CD4^+^IFN^+^ T cells (*p* < 0.0001, **Figure 5B**), CD4^+^IL10^+^ T cells (p < 0.01, **Figure 5C**) and CD4^+^IL17^+^ T cells (*p* < 0.01, **Figure 5D**).

The comparison between the groups revealed that, under the polyclonal and STAg stimulations, the GDM patients had a significantly lower percentage of CD4^+^IFN^+^ T cells (*p* = 0.0468 and *p* = 0.0267, respectively, **Figure 5B**). In addition, under STAg stimulation, GDM patients also had a significantly lower percentage of CD4^+^IL10^+^ T cells (*p* = 0.0002, **Figure 5C**) and CD4^+^IL17^+^ T cells (*p* = 0.0012, **Figure 5D**).

#### Production of cytokines by PBMCs

**Figure 6:**
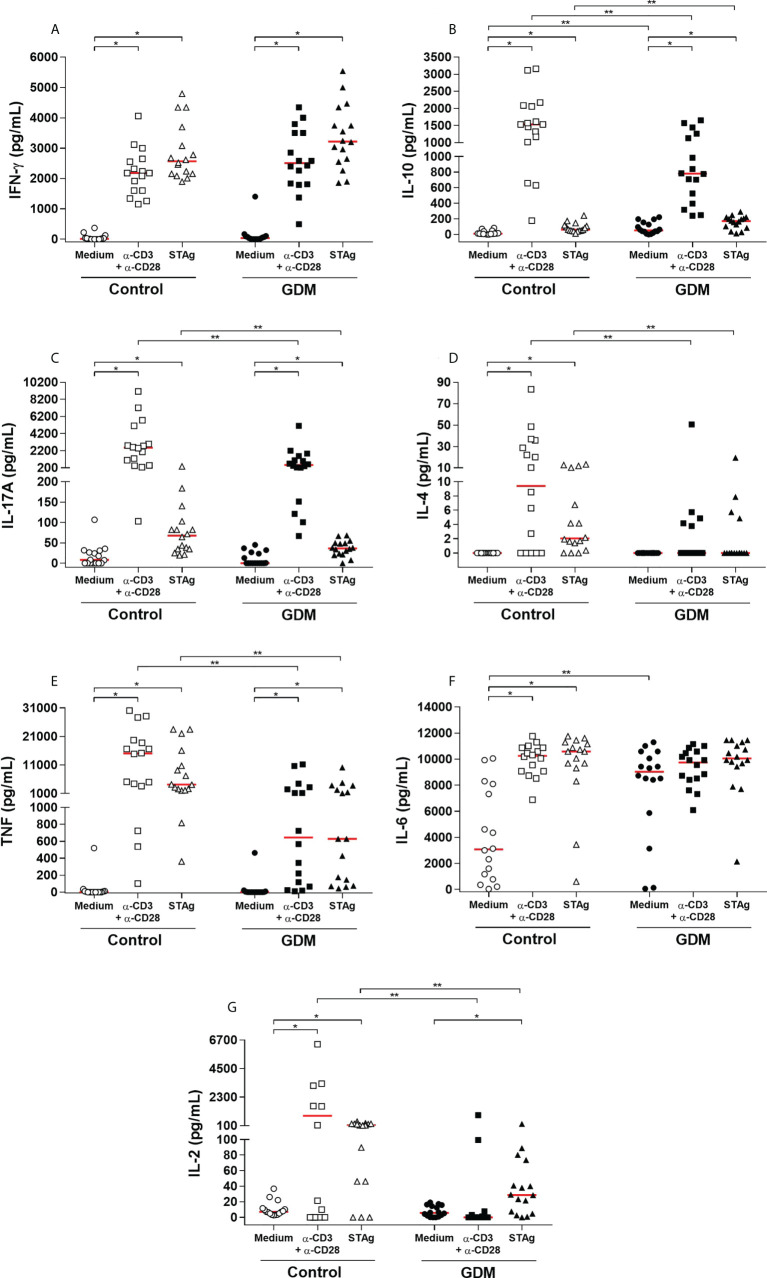
Levels of cytokines **(A)** IFN-γ, **(B)** IL-10, **(C)** IL-17, **(D)** IL-4, **(E)** TNF, **(F)** IL-6 and **(G)** IL -2, expressed in pg/mL, which were quantified by CBA in the supernatant of the three conditions of 96 hours of culture (medium, polyclonal α-CD3/α-CD28 stimulus and specific STAg stimulus) of PBMC cells from 32 pregnant women (16 GDM and 16 controls). The red line represents the median of the values. Statistically significant data, *p* < 0.05 *Wilcoxon **Mann-Whitney U test. GDM, Gestational diabetes mellitus.

Levels of IFN-γ, IL-10, IL-17, IL-4, TNF, IL-6 and IL-2 (pg/mL) of the supernatant of the three culture conditions and of the two groups of PW are shown in **Figure 6**.

In both groups, IFN-γ production was significantly higher when cells were stimulated, both with the polyclonal and STAg stimuli (*p* < 0.0001). However, there was no statistical difference when compared between the groups (**Figure 6A**).

IL-10 was also significantly higher when the cells were stimulated, with the polyclonal stimulus (*p* < 0.0001) and with STAg (*p* = 0.0003 in the GDM group and *p* < 0.0001 in the control group). In addition, the GDM group produced significantly more IL-10 than the control group when the PBMCs did not receive a stimulus (*p* = 0.0086) and in the presence of STAg (*p* = 0.0295). On the other hand, in the presence of the polyclonal stimulus, the production of IL-10 by the GDM group was significantly lower compared to the control group (*p* = 0.0077) (**Figure 6B**).

The production of IL-17 by stimulated cells was also significantly higher than by cells without any stimulus (polyclonal stimulus: *p* = 0.0001; STAg: *p* = 0.0009 GDM group, *p* = 0.0010, control group). Between the two groups, there was no statistical difference in the cells cultured with only a medium (*p* = 0.5722), but compared to the GDM group, the control group produced significantly greater amounts of IL-17, both when cells were stimulated with anti-CD3/anti-CD28 (*p* = 0.0052), and with STAg (*p* = 0.0266) (**Figure 6C**).

Only in the control group was there a significantly higher production of IL-4 by cells stimulated with anti-CD3/anti-CD28 (*p* = 0.0010) and by cells stimulated with STAg (*p* = 0.0002). In the GDM group, there was no difference between the stimuli (*p* = 0.0625 and *p* = 0.1250); there was practically no production of IL-4 by the cells of these patients. Therefore, the control patients produced significantly more IL-4 than the GDM patients, both under polyclonal stimulation (*p* = 0.0125) and under STAg stimulation (*p* = 0.0162) (**Figure 6D**).

In both groups, the production of TNF was significantly higher when the cells of the patients were stimulated, both with the polyclonal stimulus and STAg (*p* < 0.0001). Compared to the GDM group, the control group produced significantly more TNF, both under polyclonal stimulation (*p* = 0.0004) and under STAg stimulation (*p* = 0.0012) (**Figure 6E**).

The IL-6 was highly produced by the GDM group in all three culture conditions. In the control group, IL-6 production was significantly higher under polyclonal stimulus (*p* < 0.0001) and under STAg stimulus (*p* < 0.0001) when compared to cells cultured only in a medium. There was a significant difference between the groups in the production of this cytokine only in the cells cultured with a medium (*p* = 0.0064) (**Figure 6F**).

The production of IL-2 was also significantly higher when the cells were stimulated. In the GDM group, the production was significantly higher only with the STAg stimulation (*p* = 0.0010), whereas in the control group, the production was significantly higher with both the polyclonal stimulus (*p* = 0.0063), and with STAg (*p* = 0.0004). Thus, the GDM group produced significantly less IL-2 than the control group when PBMCs received polyclonal stimulus (*p* = 0.0037) and in the presence of the STAg (*p* = 0.0098) (**Figure 6G**).

### Expression of the Treg phenotype

**Figure 7:**
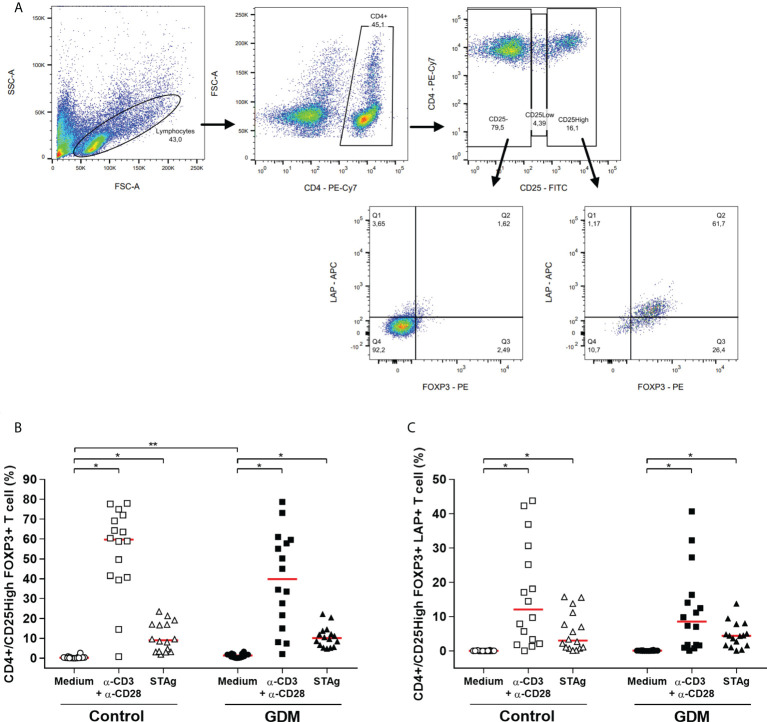
Regulatory T cell phenotype. **(A)** Gating strategy employed. Percentage of **(B)** CD4^+^/CD25^High^FOXP3^+^, **(C)** CD4^+^/CD25^High^FOXP3^+^LAP^+^ T cells, which were acquired by flow cytometry of PBMC cells from 32 pregnant women (16 GDM and 16 controls) after 96 hours of culture in three different conditions (medium, polyclonal α-CD3/α-CD28 stimulus and specific STAg stimulus). The red line represents the median of the values. Statistically significant data, *p* < 0.05 *Wilcoxon **Mann-Whitney U test. GDM, Gestational diabetes mellitus.

Through flow cytometry, it was possible to identify the phenotype of regulatory T cells (Treg) in the PBMCs of PW (**Figure 7A** - gating strategy).

Classic Treg cells are characterized as CD4^+^CD25^High^FOXP3^+^ T cells. For both PW groups, the two stimuli of the culture significantly increased the percentage of Treg cells (*p* < 0.0001). Also, CD4^+^CD25^High^FOXP3^+^ T cells were significantly higher in the GDM group in the absence of stimulation in the culture (*p* < 0.0001) (**Figure 7B**).

Additionally, the stimuli used in the culture increased the population of Treg cells that expresses LAP, in both groups (*p* < 0.0001). However, there was no difference between the groups in the number of CD4^+^CD25^High^FOXP3^+^LAP^+^ T cells (**Figure 7C**).

## Discussion

The GDM is an obstetric pathology that has been associated with an impaired maternal immune response (27), but there are few data in the literature that associate this pathology with toxoplasmosis. This study is a pioneer in evaluating how GDM can influence the maternal immune response against chronic toxoplasmosis infection.

The significantly higher glycemic averages of the GDM group in the three OGTT proved and guaranteed the distinction of the groups (GDM vs control). In both groups, the levels of education and family income were similar, but the age group of the PW with GDM was higher, and this data proves that advanced age is a risk factor for GDM development (28).

In a *T. gondii* infection in humans, all specific IgG subclasses have already been identified, whereas IgG1 is the first to be produced and the most prevalent (38–40), IgG2, IgG3 and IgG4 have variable kinetics in this type of infection (41). Our results also showed a prevalence of IgG1, followed by the IgG4 subclass in both groups, and few PW were positive for IgG2 and IgG3. Similar results were obtained in a study carried out in Mexico, which also found a prevalence of IgG1 (50%), followed by IgG4 (15%) (42).

The subclasses of antibodies present in the NBs’ serum corresponded to those found in the serum of their respective mothers. This suggests that the NBs’ antibodies were acquired *via* transplacental passage, since all four IgG subclasses cross the placental barrier, where IgG1 and IgG2 are, respectively, the subclasses more and less efficiently transported (43).

It has already been demonstrated that, at the end of pregnancy, the levels of IgG antibodies in the fetal serum, mainly IgG1, may be higher than the levels of antibodies in the mother’s serum (43, 44). This result was also obtained in the present study.

The presence of IgG antibodies in the serum of PW does not determine the time of infection precisely (45). Therefore, the IgG avidity test is widely used in the diagnosis of acute infection (46), since the higher the IgG avidity, the older the infection, which indicates a chronic toxoplasmosis; thus, low- IgG avidity is suggestive of an acute infection, indicating a larger risk of congenital transmission (47). In the present study, both groups showed high IgG avidity, indicating that all of them were indeed patients with latent toxoplasmosis infections. However, the avidity index of the GDM group was significantly lower, although without a clinically relevance. These results may suggest that these women started to produce anti-*T. gondii* antibodies by newly stimulated B cells, and that GDM interfered with the specific humoral immune response to toxoplasmosis. A study with immunosuppressed pregnant women has already shown that congenital toxoplasmosis can be transmitted by previously infected women. In these cases, a high avidity of IgG was observed with no relevant change in this clinical parameter at different measurement times (48). Previous studies have already shown that diabetes causes a reduction in the immune response (49), and the results of the three correlations in the present study (OGTT vs total IgG, IgG1 and total IgG avidity) pointed out a possible association between diabetes and the negative modulation of the specific humoral immune response against *T. gondii*. Furthermore, the glycemia at one hour after the ingestion of dextrose was the best parameter for correlation, probably as it is the time frame in which the peak of glycemia occurs.

NK cells, Th1 profile CD4^+^ T lymphocytes and cytotoxic CD8^+^ T lymphocytes have synergistic and protective activity against *T. gondii*, when occurs the differentiation for the Th2 profile, increases susceptibility to *T. gondii* (50, 51).

The immunomodulation during pregnancy contributes to the development of an environment with a predominance of Th2 and Treg cells, important for maternal immunological tolerance to the fetus, which facilitates the evasion of *T. gondii* from the immune response (52). Conversely, in pregnant women infected with T gondii, a pro inflammatory environment was associate with vertical transmission and increase newborn disease severity (53, 54).

Our results revealed that GDM did not change the amount of CD4^+^ T cells in PW. Previous studies have also found this same result (32, 55, 56), while decreases (57) and increases (58) have also been reported in the total CD4^+^ T cells in patients affected by GDM.

On the other hand, the number of CD8^+^ T cells was significantly higher in the GDM group, as well as in the presence of the specific stimulus of *T. gondii*. This result differs from the literature, whose authors did not find a difference in the number of CD8^+^ T cells between diabetic and non-diabetic PW (32, 57). However, no study has evaluated the specific cellular response to toxoplasmosis.

For T cells stimulation, it is necessary to combine several signals; the antigen-specific signal is *via* the T-cell receptor (TCR), and additional signals that are independent of the antigen occur *via* co-receptors, which define T cell destination and function. CD28 is a stimulatory co-receptor expressed constitutively on the surface of naive CD4^+^ and CD8^+^ T cells (59), while PD-1 is an inhibitory co-receptor (60). Inhibitory co-receptors play an important role against the unrestricted activation of T lymphocytes, helping to maintain peripheral tolerance and immunological homeostasis during an infection (59, 61).

In this study, without stimulation, the patients with GDM had a greater number of CD4^+^PD-1^+^ T cells, and this difference was also previously found (32). It is known that the activation of PD-1 suppresses the functions of T lymphocytes through the dephosphorylation of TCR signaling components (59), however, it was demonstrated that PD-1 prefers the signaling of the CD28 co-receptor as a target for dephosphorylation (61). Therefore, the cells expressing the double labeling of CD28 and PD-1 are likely to have the most inhibitory effect when PD-1 is activated by their ligand. Thus, the results of the present study demonstrate that the cells of the GDM patients were more activated and taken to exhaustion, since the number of CD8^+^ and CD4^+^ T cells concomitantly expressing CD28 and PD-1 was significantly greater in the GDM group.

CD8^+^ T lymphocytes play a fundamental role in chronic toxoplasmosis infection control (62, 63), as they are important for maintaining *T. gondii* in quiescence and preventing the reactivation of latent infection (64). A progressive increase of PD-1 expression in the CD8^+^ T cells of mice during the chronic phase of infection by *T. gondii* was correlated with parasitemia reactivation (65). Therefore, increased PD-1 is indicative of cells in exhaustion, with loss of function in controlling infection and consequently, the risk of acute disease reactivation. The data from this study showed that the GDM group had more CD8 T cells expressing PD-1, suggesting the potential risk for this group.

CD4^+^ T cells are necessary for the maintenance of CD8^+^ T functions (62). In the absence of CD4^+^ T, cytotoxic lymphocytes cannot perform their role well since the generated immunity cannot be maintained and the response to a secondary challenge becomes weak (63). During chronic *T. gondii* infection, specific CD4^+^ T cells increase the expression of the transcription factor B-lymphocyte-induced maturation protein (BLIMP-1), with a consequent increase in inhibitory receptors, including PD-1, and loss of functionality. Thus, CD4^+^ T cells in exhaustion do not adequately help CD8^+^ T cells to effectively function against chronic toxoplasmosis infection (65). Our results showed that the GDM group had more CD4^+^PD1^+^ T cells, therefore suggesting that the response of T helper lymphocytes in diabetic patients is also impaired.

Additionally, it is known that the binding of PD-1 to its ligands results in the inhibition of lymphocyte proliferation and cytokine secretion (60, 67, 68). These data corroborate partially with the results obtained in the present study, wherein the GDM patients had more T lymphocytes expressing PD-1 and also secreted less IL-10, IL-17, IL-4, TNF and IL-2 cytokines.

The IL-6 was highly produced by the PBMCs of the GDM group. High secretion of IL-6 participates in the pathogenesis of GDM, as it can aggravate insulin resistance in pregnancy (69). In comparison with non-diabetic PW, the increase in serum levels of IL-6 in GDM patients has already been reported in several studies (69–72).

Some authors found that the serum levels of IL-4 were not different between women with and without GDM (58, 70). In the present study, when stimulated, the PBMCs of the GDM group practically did not produce IL-4 while the control group produced significantly more IL-4. This suggests that the Th2 profile of the diabetic patients was lower than the control patients.

IL-2 promotes the expansion and survival of T cells, so their removal can lead to cell death (60), and it has already been demonstrated that TNF-α plays an important role in chronic *T. gondii* infection (73, 74). The PBMCs of the GDM group secreted significantly less IL-2 and TNF than the control group. Both are Th1 profile cytokines, so this suggests that GDM also negatively modulates the Th1 profile of PW’s cells, including in the specific response to toxoplasmosis.

IFN-γ is a characteristic cytokine of the Th1 profile, produced in response to intracellular infections, with fundamental importance in toxoplasmosis control (50, 51, 75, 76). However, literature data vary as it has been reported that the serum IFN-γ concentrations of PW with GDM were similar (58), lower (70), and higher (77) than normoglycemic patients. In this study, the evaluation of IFN-γ-producing CD4^+^ T cells revealed that patients with GDM had a significantly lower percentage of CD4^+^IFN^+^ T cells, therefore, a decrease in CD4^+^ T cells of the Th1 profile. In contrast, the quantification of IFN-γ in the culture supernatant revealed that there was no difference in the secretion of this cytokine between the PBMCs of the two groups of PW. This suggests that other mononuclear cells from diabetic patients, such as monocytes and/or CD8^+^ lymphocytes and/or NK cells, should be more activated to help control toxoplasmosis *via* the production of this pro-inflammatory cytokine. Hara et al. (77) showed that there is a greater amount of NK cells in the blood of PW with GDM.

Patients with GDM also had less Th17 cells (CD4^+^IL17^+^ T cells). Furthermore, IL-17 secretion by PBMC was also significantly lower in the GDM group. No other study investigated the Th17 cell profile and production of IL-17 in response to toxoplasmosis in GDM patients; what has already been reported is that there is no difference between the amount of IL-17 in the plasma of GDM and controls PW (56, 78), and these results are similar with the present study, for the culture condition without stimulation.

IL-10 is necessary for the survival of the host both in the acute and chronic phases of *T. gondii* infection (79). The analysis of IL-10-producing CD4^+^ T cells revealed that patients with GDM had a significantly lower percentage of CD4^+^IL-10^+^ T cells specific for the *T. gondii* antigen. Contradictorily, in the culture supernatants of the GDM group, PBMCs secreted significantly more IL-10 than the cells of the control group, both when they did not receive stimulus and in the presence of the STAg stimulus. These results are compatible with the literature, in which Atègbo et al. (70) found that serum levels of IL-10 were significantly increased in the women with GDM compared to the control women. Therefore, this data suggests that other peripheral blood cells are contributing to the high levels of IL-10 in the supernatant of the patients with GDM. IL-10 can be produced by multiple cell types of the innate and adaptive immune system, such as eosinophils, DC, macrophages, NK cells, B lymphocytes, CD8^+^ T lymphocytes and several CD4^+^ T cell subtypes, for example, regulatory helper T lymphocytes (Treg) (80).

The Treg cell plays an important role in maintaining a healthy pregnancy; once the embryo expresses alloantigens derived from the father, a tolerogenic maternal immune response, mediated by Treg, is necessary. Thus, during pregnancy, the number of Treg cells is increased through many mechanisms, while the decrease or dysfunction of these cells is associated with women’s infertility, recurrent abortions or pregnancy complications (81). Treg cells are also necessary for the maintenance of pregnancy after *T. gondii* infection (82).

The Treg subpopulation is characterized as a CD4^+^ T cell that expresses high amounts of CD25 on its surface (CD25^High^) and has the forkhead box P3 (FOXP3^+^) as its transcription factor. Activated and functional Treg cells, which have better immunoregulatory effects, express the latency-associated peptide (LAP) protein on their surface (83–85).

In this study, STAg stimulation similarly influenced the amount of Treg in the two PW groups, and there was no difference between the GDM and control groups in the number of CD4^+^CD25^High^FOXP3^+^LAP^+^ Treg cells. All PW involved, diabetic and non-diabetic, had a good pregnancy until the end, suggesting that the Tregs were functional in both groups.

**Figure 8:**
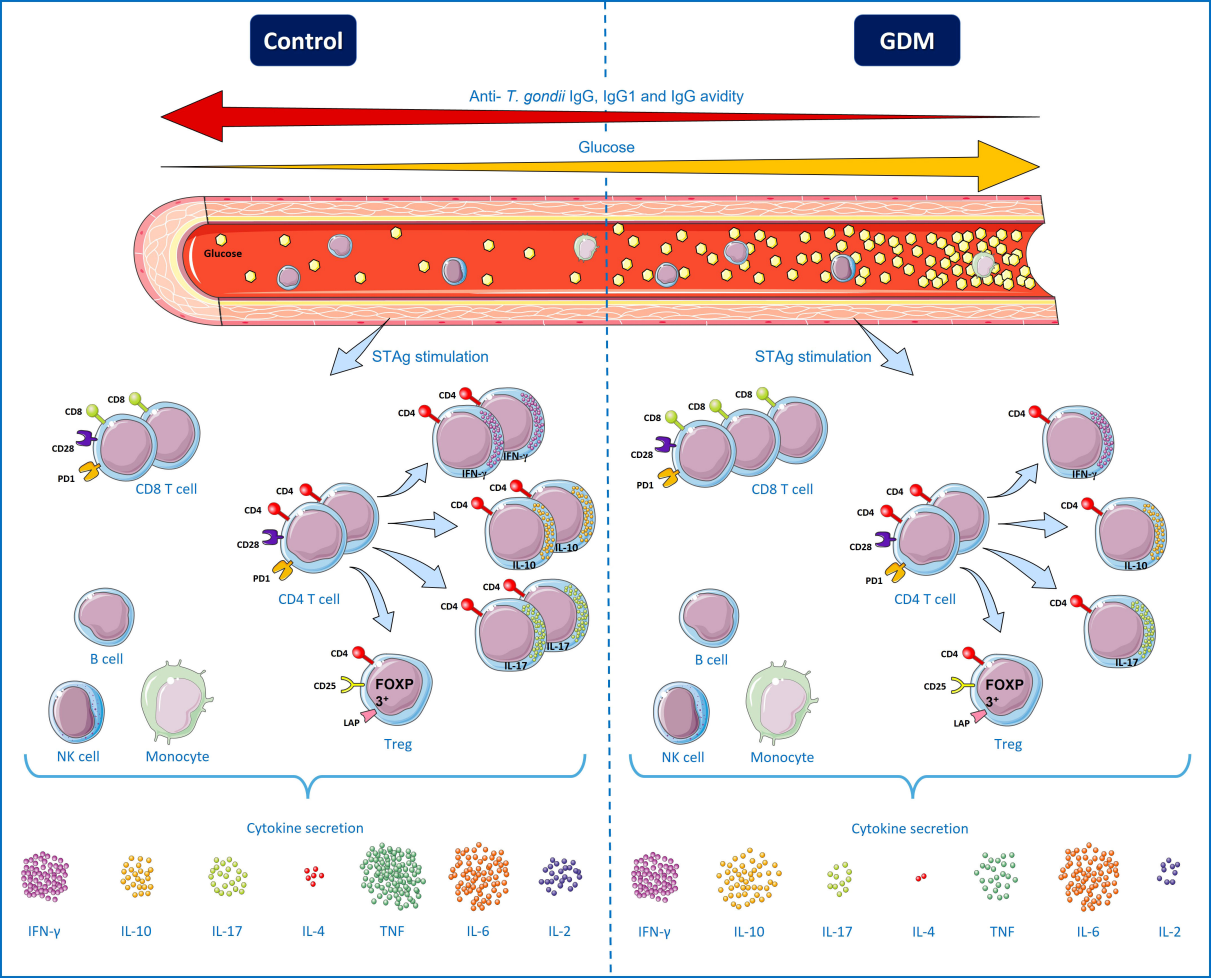
Specific *T. gondii* immune responses from pregnant women chronically infected by the parasite, with and without gestational diabetes mellitus (GDM). High blood glucose levels characterize patients with GDM, while normoglycemic patients were identified as patients in the control group. There was a negative correlation between the blood glucose of PW and the levels of total IgG, IgG1 and IgG avidity, therefore, the higher the patient’s glycemia, the lower the level of anti-*T. gondii* antibodies. Under polyclonal stimuli (anti-CD3/anti-CD28), the GDM group patients had a higher number of T lymphocytes expressing the markers of cell activation and exhaustion (CD28 and PD-1), but in the presence of STAg, there was no difference. Under STAg stimuli, the number of CD8^+^ T cells was higher in the GDM group, while the number of CD4^+^ T cells was similar between the groups. However, the amount of CD4^+^ T lymphocytes producing IFN-γ, IL-10 and IL-17 was significantly lower in the GDM group, while there was no difference between the groups in the amount of functional CD4^+^CD25^High^FOXP3^+^LAP^+^ Treg cells. IL-10 was secreted more by peripheral blood mononuclear cells (PBMC) of the GDM patients, there was no difference between the groups in the secretion of IFN-γ and IL-6, while the secretion of cytokines IL-17, IL-4, TNF and IL -2 was lower in the GDM group. Therefore, compared to normoglycemic PW, PW who develop GDM have a decrease in the responses of the Th1, Th2 and Th17 profile, as well as a decrease in IgG avidity and a correlation between the increase in blood glucose and the decrease in anti-*T. gondii* antibodies, suggesting an association between GDM and the negative modulation of cellular and humoral immune responses against *T. gondii*. B cells, NK cells and monocytes are separated by a box, to represent that they are also PBMCs, but they were not directly analyzed in this work. Terms of use: This figure is derived from images attributed to Servier’s “Servier Medical Art”, used under the Creative Commons Attribution 3.0 France (CC BY 3.0 FR) license terms.

All the results presented suggest that, compared to normoglycemic PW, patients who developed GDM had a decreased immune response in the Th1, Th2 and Th17 profile, while the Treg profile was not affected in STAg stimulated cultures. Furthermore, patients with GDM also had a decrease in IgG anti-*T. gondii* avidity, and demonstrated a correlation between an increase in blood glucose levels and decrease in IgG avidity, total IgG and IgG1 (**Figure 8**). Thus, it is possible to conclude that GDM negatively modulates the humoral and cellular immune response of PW chronically infected with *T. gondii*.

In chronic toxoplasmosis, tissue cysts of the parasite usually remain inactive for the host’s whole life, but tachyzoites can re-emerge and cause disease/symptoms in some patients, usually associated with suppression of the host’s immune response, but the mechanisms of reactivation have not been well described (62).

Since GDM can modulate the PW’s immune response, it is important to closely monitor the patients considered to be immune to toxoplasmosis and their newborns, with more studies to assess the real possibility of reactivation of the tissue cysts at the end of pregnancy, due to the influence of diabetes. This is of great importance because when the congenital infection caused by *T. gondii* occurs in the third trimester of pregnancy, the late transmission of parasites reduces the time for the parasite to multiply in the fetus or newborn, and consequently, the damage is not evident early. For this reason, congenital infections are often neglected because they are asymptomatic at birth and remain unnoticed. However, clinical manifestations may appear weeks or years after birth (86), usually in the form of severe chorioretinitis (87).

## Data availability statement

The raw data that support the conclusions of this article will be made available upon request.

## Ethics statement

The study was reviewed and approved by Universidade Federal do Triângulo Mineiro’s Ethical Committee (Protocol number: 1.870.741). The patients/participants provided their written informed consent to participate in this study.

## Author contributions

AO-S was involved in the sample collection, carried out all the laboratory tests, performed the statistical analysis, participated in the design of the study and drafted the manuscript. PF and RR participated in the sample collection and laboratory testing. CR-A was involved in the parasite maintenance in the cell culture and antigen preparation. AG and MP participated in the design of the study and data analysis. FV, TF-d-A and MS were involved in the flow cytometry and statistical analysis. JM, DR, VR were the researchers responsible for the experimental design, coordination of the study, data analysis and revision of the manuscript. All authors contributed to the article and approved the submitted version.

## Funding

The financial support received from FAPEMIG allowed the acquisition of equipment and maintenance of the Laboratory of Immunology. CAPES and CNPq grants were applied to the maintenance of the Laboratory of Immunology. Publication fees will be paid with resources from CNPq and CAPES.

## Acknowledgments

The authors thank all Gynecology and Obstetrics Clinical employees for technical assistance. This study was supported by Brazilian Funding Agencies (CNPq, FAPEMIG – REDE 313-16).

## Conflict of interest

The authors declare that the research was conducted in the absence of any commercial or financial relationships that could be construed as potential conflicts of interest.

## Publisher’s note

All claims expressed in this article are solely those of the authors and do not necessarily represent those of their affiliated organizations, or those of the publisher, the editors and the reviewers. Any product that may be evaluated in this article, or claim that may be made by its manufacturer, is not guaranteed or endorsed by the publisher.
